# Label-free blood cell separation for space health monitoring using a portable blast cell biochip

**DOI:** 10.1038/s41526-026-00561-9

**Published:** 2026-01-27

**Authors:** Martina Mugnano, Vincenza Cerbone, Massimiliano Maria Villone, Annalaura Montella, Giulia Scalia, Mario Capasso, Achille Iolascon, Lisa Miccio, Pietro Ferraro, Silvia Mari, Francesca Ferranti, Pier Luca Maffettone

**Affiliations:** 1 Department of Chemical, Materials and Production Engineering, University of Naples Federico II, Napoli, Italy; 2 CEINGE—Advanced Biotechnologies, Napoli, Italy; 3 DMMBM, Department of Molecular Medicine and Medical Biotechnology, University of Naples ‘Federico II’, Napoli, Italy; 4 ISASI – Institute of Applied Science and Intelligent Systems – National Research Council (CNR), Pozzuoli, Italy; 5 Italian Space Agency (ASI), Rome, Italy

**Keywords:** Biological techniques, Biotechnology, Cancer, Engineering

## Abstract

We demonstrate a novel biomedical application of a commercial spiral microfluidic chip (Fluidic 382), originally developed for particle sorting, by repurposing it for label-free, size-based isolation of pathological blood cells, including leukemic blasts from acute myeloid leukemia (AML) samples. For the first time, we establish and validate a streamlined protocol for cell separation using Dean-driven hydrodynamic forces in a chip not originally designed for blood analysis. Using a 9-turn, 6-outlet spiral channel configuration, we achieved high-efficiency sorting of red and white blood cells from healthy donors and selectively enriched pathological blasts from AML patient blood. The device’s performance was validated through flow cytometry and numerical simulations, demonstrating strong agreement between experimental and computational results with less than 1% relative error. With its compact footprint, reagent-free operation, and automation potential, this method represents a significant advance toward point-of-care blood diagnostics in extreme environments, particularly space missions. The chip’s ability to separate pathological cells in microgravity-compatible conditions offers a promising route for real-time astronaut health monitoring, supporting early detection and mitigation of radiation-induced haematological disorders such as AML.

## Introduction

In recent years, cell separation using microfluidics has revolutionized biomedical analysis techniques, offering rapid, portable, and label-free solutions for isolating cells from complex samples such as blood^1^. In particular, spiral devices exploit hydrodynamic forces to efficiently separate cells based on size and deformability, representing a valid alternative to traditional methods, which are often bulky and dependent on fluorescent markers see Table 1^2–5^.

**Table 1:** Advantages and disadvantages of FACS and spiral chip for space applications

Technology	Advantages	Disadvantages	References
**Spiral Chips**	• Compact and portable • Low energy consumption • Easy automation • High efficiency and throughput • Less cell stress • Suitable for continuous and rapid separation	• Selection based mainly on size/deformability (less specific) • Limited multiparametric selection • Technical optimization still ongoing	[^2, 3^]
**FACS**	• High accuracy and specificity • Multiparametric selection • Widely used and validated in research	• Bulky and complex • Requires skilled personnel • High energy consumption and maintenance • Difficult to miniaturize for space use	[^4, 5^]

Spiral chips are especially promising for space applications due to their compactness, ease of automation, and low resource requirements, which are critical in space environments. Fluorescence-Activated Cell Sorting (FACS), although is highly precise, is less suitable for space missions because of its size, complexity, and maintenance needs. Efficient separation of blood cells is fundamental for early diagnosis and monitoring of hematological diseases, such as acute myeloid leukemia (AML), and for applications in resource-limited environments, including space missions. Spiral microfluidic devices, through the generation of Dean and hydrodynamic lift forces, enable label-free separation of cells with different sizes and rigidity, such as red blood cells (RBCs), white blood cells (WBCs), and pathological cells (blasts)^1,6–9^. These systems offer advantages such as high recovery efficiency, rapid analysis, and minimal sample preparation, making them ideal for point-of-care applications and use in extreme conditions.

The growing need for rapid, reliable, and minimally invasive diagnostic tools in both clinical and extreme environments has accelerated the demand for portable, automated blood analysis platforms. In conventional healthcare settings, access to flow cytometry or cell sorting instruments is often limited by infrastructure, cost, and operator expertise—constraints that are even more pronounced in remote or resource-scarce regions. This challenge is further amplified in spaceflight, where microgravity, limited crew training, and strict payload constraints preclude the use of traditional laboratory equipment^10^. The ability to perform label-free, real-time blood cell separation directly from peripheral blood samples could enable on-the-spot detection of immunological shifts or pathological signatures, such as leukemic blasts, without the need for complex processing or staining. Therefore, translating spiral microfluidic technology—originally developed for laboratory-scale particle sorting—into a field-deployable biomedical diagnostic tool represents a critical step toward personalized, autonomous health monitoring in both terrestrial and extraterrestrial settings.

Indeed, the use of microfluidic chips for blood cell separation is an emerging technology with enormous potential for space applications. During space missions, astronauts face unique challenges such as exposure to microgravity, space radiation, and prolonged isolation, which can significantly impact the immune system and overall health.

Microfluidic chips provide a compact, efficient, and precise method for separating blood components, such as red blood cells, white blood cells, and plasma. This separation is essential for real-time monitoring of astronauts’ health by analysing critical biomarkers for immunity, metabolism, and cellular regeneration. Furthermore, microfluidic devices are ideal for the space environment as they consume minimal reagents, require very small sample volumes, and can be easily integrated into automated systems, such as optical sensors^11^.

Spiral microfluidic chips represent a promising technology for hematological diagnostics in microgravity, offering label-free, high-efficiency separation of blood cells based on size and deformability. Their compact design, minimal reagent requirements, and automation potential make them particularly suitable for aerospace applications, where traditional laboratory equipment is impractical or impossible to use^12^. In microgravity environments, spiral geometries leverage hydrodynamic forces—such as Dean flow—to achieve continuous, high-throughput cell sorting without the need for bulky instrumentation or complex sample preparation. Recent studies have demonstrated that these devices can efficiently isolate and analyze various blood cell populations, supporting real-time, point-of-care diagnostics essential for astronaut health monitoring during space missions. The adaptability and robustness of spiral microfluidic platforms thus provide a critical foundation for autonomous biomedical monitoring and early detection of hematological disorders in extreme environments, including long-duration spaceflight^13^.

As space exploration expands beyond Earth’s orbit, the health risks posed by prolonged exposure to the unique environment of space have become a critical focus of research. One of the primary concerns is the impact of ionizing radiation, which astronauts are exposed to at much higher levels than on Earth. Ionizing radiation in space comes from various sources, including galactic cosmic rays (GCRs), solar particle events (SPEs), and trapped radiation in Earth’s magnetosphere^14^. Exposure to ionizing radiation can occur in both acute and chronic forms^15^. Acute doses—such as those that may result from intense solar particle events—deliver high levels of radiation over a short period, potentially causing immediate biological damage and significantly increasing cancer risk^16,17^. Chronic doses, by contrast, represent continuous low-dose exposure over extended durations, as would occur during long-term space missions. While less immediately damaging, chronic exposure still poses a cumulative risk of cellular mutations and malignancies over time^18^. Prolonged exposure to such radiation has been shown to induce cellular damage, mutations, and an increased risk of developing cancer^19–24^. Among the haematological malignancies, AML is of particular interest due to its known sensitivity to ionizing radiation^20,25^. AML is a rapidly progressing cancer that affects the bone marrow’s ability to produce normal blood cells, leading to an accumulation of immature white blood cells^26^. Epidemiological studies on Earth have established a significant association between ionizing radiation exposure, particularly from medical procedures or nuclear events, and the development of AML^27,28^. These findings align with historical data from atomic bomb survivors, which show an elevated incidence of leukaemia following radiation exposure^29–31^. However, the type and intensity of radiation encountered in space present unique challenges and risks that remain poorly understood. As interest in long-duration missions to destinations like Mars or lunar bases grows, it becomes imperative to assess the potential correlation between space radiation exposure and AML incidence. This assessment is crucial for developing effective protective measures for astronauts^32,33^.

**Fig. 1:**
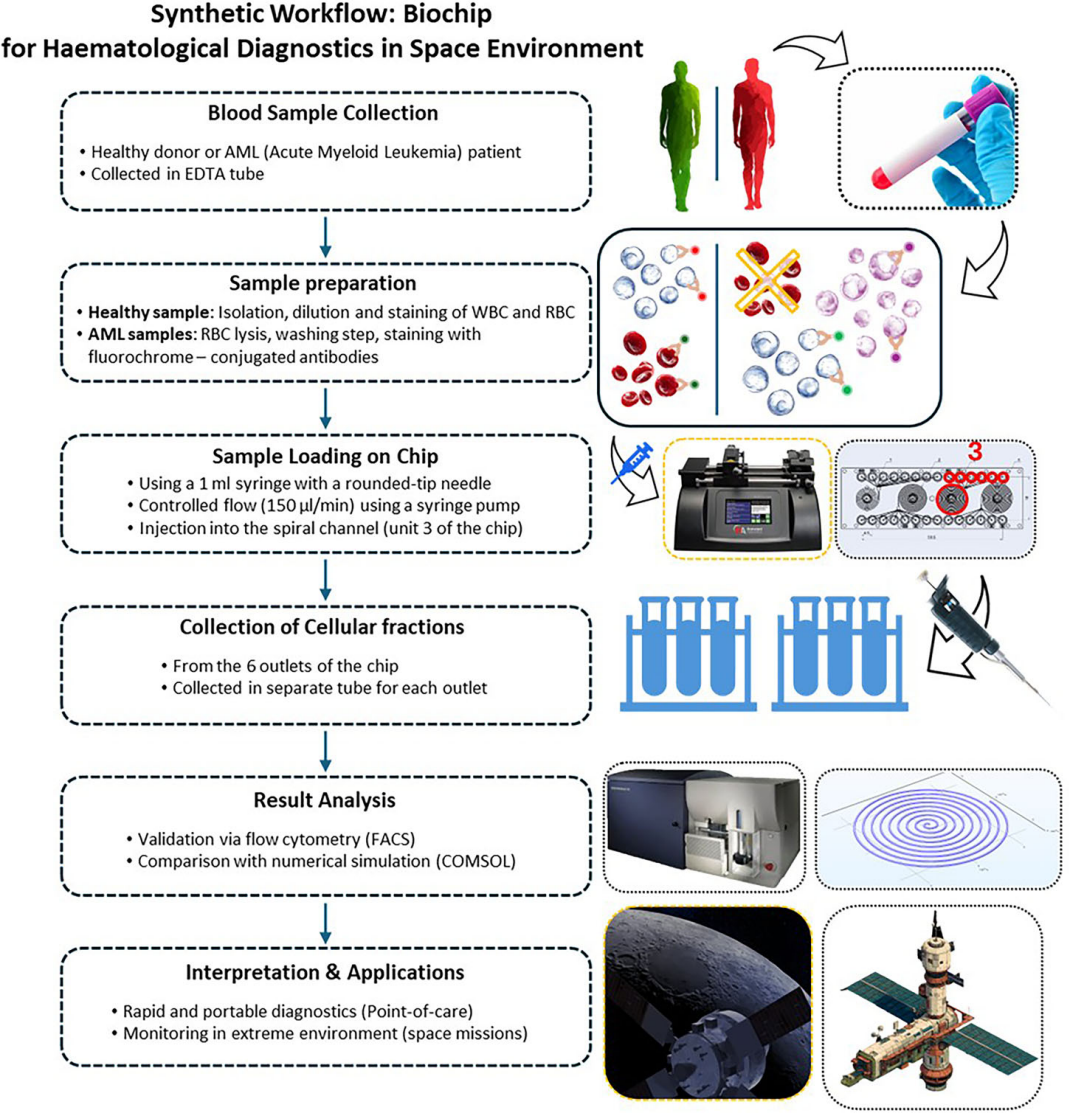
Schematic representation of the proposed biochip procedure for hematological diagnostics in a space environment, illustrating the main steps of sample processing and analysis. Schematic workflow of the proposed procedure.

In this study, we evaluate the performance of a spiral microfluidic chip for the separation of blood cells from both healthy and pathological samples (AML, FAB M5 subtype), comparing experimental results with numerical simulations. The aim is to demonstrate the effectiveness and precision of the system for advanced diagnostic applications, with particular attention to the selective separation of pathological cells in contexts where portability and operational autonomy are essential. In particular, in this paper, a detailed procedure for blood analysis using spiral microfluidic devices is presented that allows size-based isolation of blood cell populations using hydrodynamic forces present in curvilinear microchannels. A schematic workflow is shown in Fig. 1. Biological samples from multiple healthy donors and AML patients were included in the study. Informed consent was obtained from all participants in accordance with institutional and ethical guidelines. The experimental section was supported by numerical simulation. Spiral chip sorters are a type of microfluidic device used for the isolation and separation of cells. They rely on the principles of centrifugal force and hydrodynamic effects to separate cells based on their size, shape, and other physical characteristics^34^. In the context of isolating circulating tumour cells (CTCs), spiral chip sorters have gained significant attention as a promising tool for early cancer detection and monitoring. CTCs are cells that have detached from a primary tumour and entered the bloodstream, where they can be carried to other parts of the body and potentially form new tumours^35,36^. Detecting and analysing CTCs can provide valuable information about the status of a patient’s cancer and guide treatment decisions^37^. The possibility of fast-processing time and the ability to collect and sort blood populations and to detect possible pathological cells from a small patient blood volume would open in future to a broad range of biomedical applications, for example in space field. Indeed, there is a great demand of new technologies to monitor the human health during space missions avoiding complex sample preparations and suppling reliable results without highly skilled personnel and cumbersome tool such as cell sorter machine. In summary, these chips represent an advanced solution for diagnosing and managing astronauts’ health, contributing to mission safety and the development of innovative technologies for terrestrial applications.

## Results

### Experimental characterization of a microfluidic device for cell sorting

**Fig. 2:**
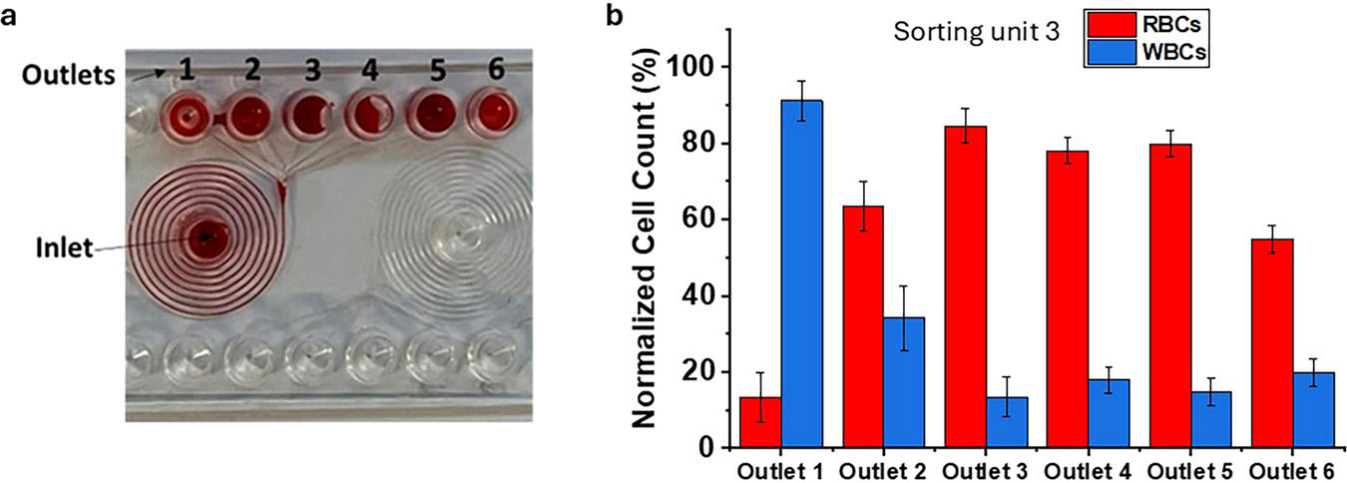
Results of sorting unit 3 for RBC and WBC sorting. **a** Picture of the spiral chip with a blood sample, illustrating the configuration of the sorting unit. **b** The results of sorting unit 3 for RBC and WBC sorting.

Figure 2 presents the experimental results of a microfluidic device featuring 9 spiral channels and 6 outlet wells, designed to separate RBCs, (size 7 µm) from WBCs, (size 10–15 µm). This configuration is conventionally referred to as sorting unit 3. Below is an in-depth analysis based on the details provided. Device Design: the sorting unit consists of 9 spiral channels with a channel width of 150 µm and a depth of 70 µm. The design leverages the centrifugal forces generated by the spiral flow to separate cells based on their size and deformability. The separation was performed at a flow rate of 150 µl/min, sufficient to drive the cells along the channel without compromising their structural integrity. Regarding the red blood cells (RBCs): a positive trend was observed from well 1 to well 3, indicating that the smaller RBCs progressively accumulate in the outlets farther from the inlet point. WBCs showed an opposing trend, with higher concentrations in well 1. This suggests that the larger size and rigidity of WBCs make them more likely to deviate toward the nearest outlet. In the well 1 the separation efficiency exceeding 90% for WBCs, confirming that most of these cells were isolated in this section. In the well 3 an efficiency of over 80% for RBCs was achieved, indicating a high capacity to isolate these cells in the expected regions. The differential distribution highlights that the microfluidic technology reliably separates cells of varying sizes and properties through hydrodynamic forces. The channel configuration, coupled with precise control of the flow rate, enables accurate sorting without the need for chemical reagents or fluorescent markers. This makes the system ideal for autonomous and reproducible applications, such as in space environments, where bulky and sophisticated machinery like FACS would be impractical. These findings validate the potential of this device for advanced biomedical applications, particularly where portability, precision, and efficiency are critical.

### Analysis of acute myeloid leukemia blood sample using microfluidic sorting

**Fig. 3:**
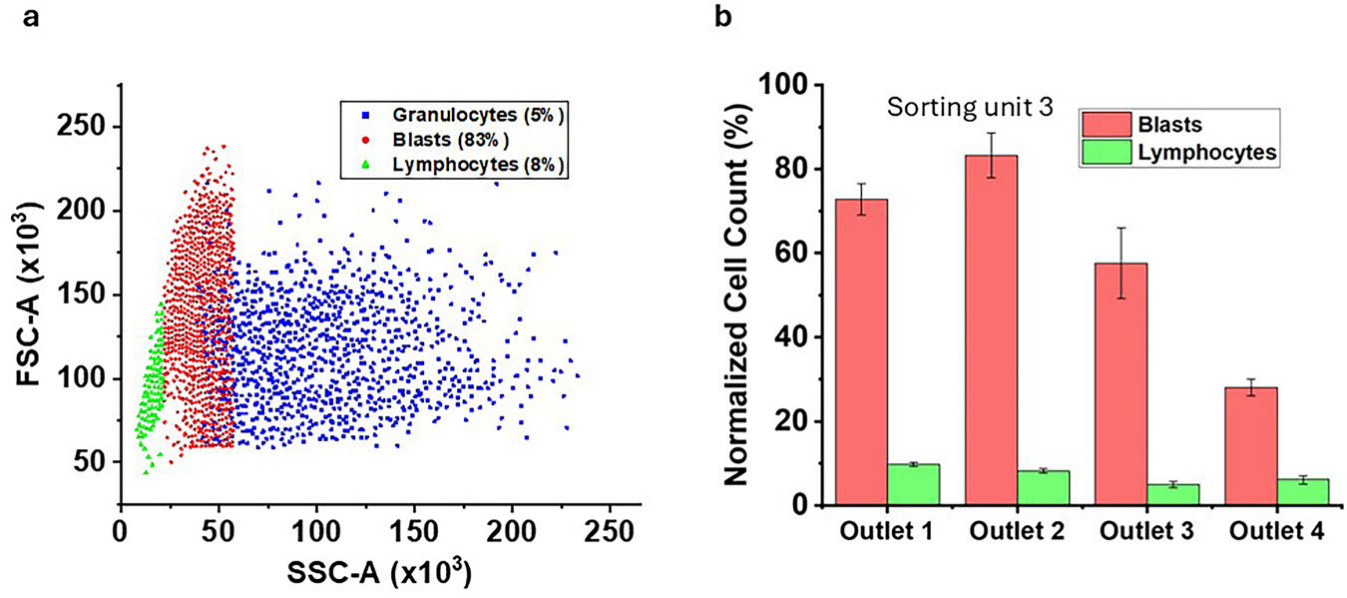
Microfluidic separation of pathological blood cells from an acute myeloid leukemia sample. **a** Forward vs side scatter plot of blood with AML. Red dots represent blasts cells. Green dots are lymphocytes and blue represent granulocytes (**b**) Results of sorting unit 3 for patient with AML. Negative trend of pathological cells from outlet 1 to outlet 4. 83% separation efficiency for pathological cells (10–17 μm) at outlet 2.

Figure 3 presents the results of an experimental campaign involving a pathological blood sample from three patients with AML, each of which was analysed in triplicate to minimize experimental variability, for a total of nine measurements. The samples with a comparable leukocyte count contain approximately 83% pathological cells (blasts, size ~15 µm), which are larger than typical white blood cells; 8% lymphocytes (size 7–8 µm); and 5% granulocytes (size10–15 µm). The image illustrates the outcomes of a microfluidic sorting experiment targeting a blood sample affected by AML. Specifically, according to French-America-British classification, the sample is classified as FAB LM5, a monoblastic leukemia characterized by large blasts^38^.

Scatter Plot Analysis (Fig. 3a): the Forward Scatter (FSC) vs. Side Scatter (SSC) plot provides insights into the size and granularity of the sample’s cellular components:

- Red dots (pathological cells – blasts): Concentrated in a specific region of the graph, characterized by high FSC values, indicating their large size.
- Green dots (lymphocytes): Located in areas with low FSC and SSC values, consistent with their smaller size (7–8 µm) and low granularity.
- Blue dots (granulocytes): Exhibit cellular heterogeneity, displaying higher scatter values compared to other cell types.

This graph confirms the sample composition:

- 83% blasts (pathological cells), the primary contributors to AML.
- 8% lymphocytes, representative of the healthy immune system.
- 5% granulocytes.

The pathological samples included in this study serves as a proof-of-principle demonstration of the workflow we established. At this stage, expanding the analysis to multiple pathological samples with comparable leukocyte differential counts and blast percentages is challenging due to the substantial biological variability among leukemia patients and the limited availability of clinically similar samples. Nevertheless, the validation of this workflow in a larger, homogeneous patient cohort remains a crucial future objective. Planned studies will analyse additional samples with similar hematological characteristics to enable more robust statistical comparisons and further validate the experimental approach. In this context, in Table 2 is reported the distribution of RBC, WBC, blasts, and lymphocytes at the three device outlets for samples from healthy donors and AML patients. Values are reported as mean ± standard deviation. Different letters (a–c) denote statistically significant differences among outlets for the same cell type (*p* < 0.05).

**Table 2:** Statistical analysis, mean ± standard deviation of cell distribution (%) at the three device outlets for samples from healthy donors and AML patients

Patient	Cell type	Outlet 1*	Outlet 2*	Outlet 3*
Healthy	**RBC**	13.24 ± 6.51 (c)	63.40 ± 6.50 (b)	84.60 ± 4.61 (a)
	**WBC**	91.15 ± 5.12 (a)	34.13 ± 8.40 (b)	13.32 ± 5.21 (c)
AML	**BLASTS**	72.83 ± 3.75 (b)	83.23 ± 5.35 (a)	57.58 ± 8.38 (c)
	**LYMPHO**	9.77 ± 0.50 (a)	8.18 ± 0.56 (b)	4.96 ± 0.72 (c)

Different letters (a–c) indicate statistically significant differences among outlets for each cell type (*p* < 0.05).

*Different letters means significantly different groups.

In Fig. 3b is reported the distribution of cells exiting the four wells of the microfluidic sorting unit, focusing on blasts and lymphocytes:

- Blasts (blue columns): Display a negative trend from well 1 to well 4, with the highest concentration (83% separation efficiency) in well 2.
- Lymphocytes (green columns): Evenly distributed across the four wells, consistent with their low percentage in the sample.

Regarding blast separation: The large size of blasts (15 µm) makes them more prone to deviation into the first wells (1 and 2) due to hydrodynamic and centrifugal forces within the microfluidic channel. The 83% separation efficiency in well 2 highlights the device’s ability to selectively isolate pathological cells, potentially enabling rapid and accurate diagnostics or targeted treatments. Concerning lymphocyte distribution: the uniform distribution across the wells reflects the low proportion of lymphocytes in the sample (~8% compared to 83% blasts). This pattern underscores the performance of the spiral microfluidic chip in isolating and concentrating larger pathological cells while maintaining the integrity of smaller, healthy cells. The results demonstrate the effectiveness of the microfluidic device in isolating and analysing pathological cells in an AML sample. Its ability to achieve high separation efficiency for large blasts and uniform distribution for smaller lymphocytes suggests its potential for precise and portable biomedical applications, particularly in settings requiring rapid analysis or minimal equipment. For both the control and pathological samples, the separation efficiency (%) was defined as the recovery efficiency, calculated as the ratio between the number of target cells recovered at the expected outlet and the total number of target cells introduced into the device. The obtained mean efficiency for the pathological sample (~83) is very close to the recovery rates typically reported for standard FACS Aria III cell sorting, which achieves >98% purity and >80% yield of Poisson’s expected value under standard four-way sorting conditions (70 psi, 90 kHz), highlighting the strong performance of the spiral chip under label-free conditions.

### Numerical simulation

To support the experimental findings shown in Fig. 3, we performed a numerical simulation of cell sorting with commercial software COMSOL Multiphysics V6.2. In particular, we employed the Laminar Flow and Particle Tracing for Fluid Flow packages.

**Fig. 4:**
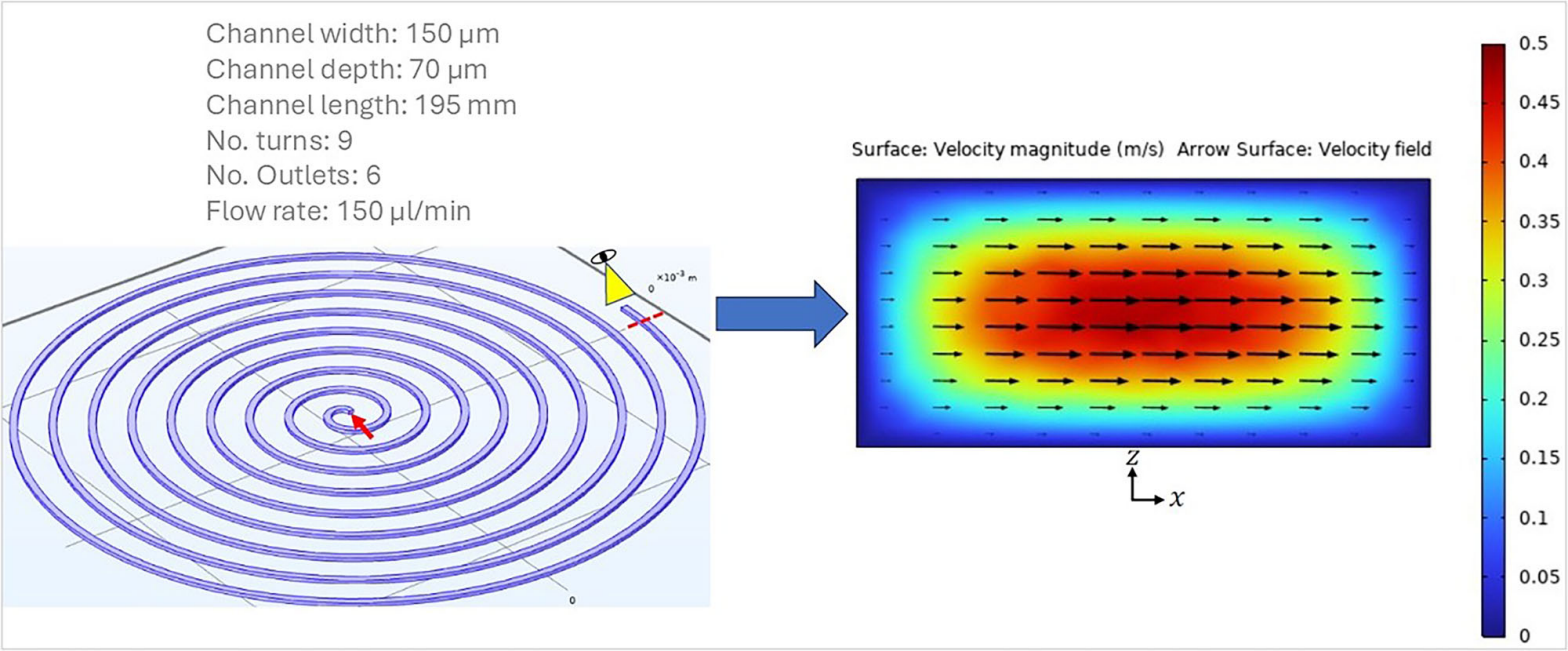
Numerical simulation of cell separation through Dean flows. Geometry of the microfluidic channel considered in the simulation with specification of dimensions and fluid flow rate (left) and map of fluid velocity magnitude and vector field of transversal fluid velocity on the cross section of the channel identified by the red dashed line on the left (right).

**Fig. 5:**
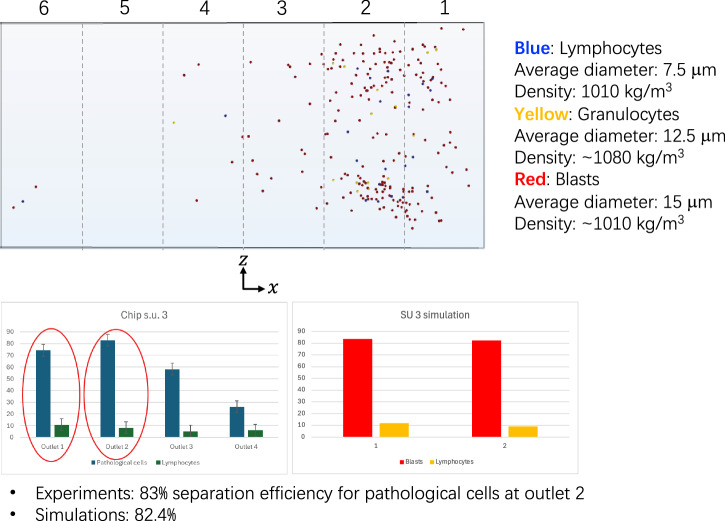
Numerical simulation illustrating the separation of cells through Dean flows, showing the trajectories and distribution of different cell types within the microfluidic channel. Distribution of the three “families” of cells considered in the simulations throughout the outlet section of the channel (top, see on the right for the geometrical and physical parameters) and comparison between the experimental (left) and numerical (right) distributions of the cells in outlets 1 and 2 (bottom).

**Fig. 6:**
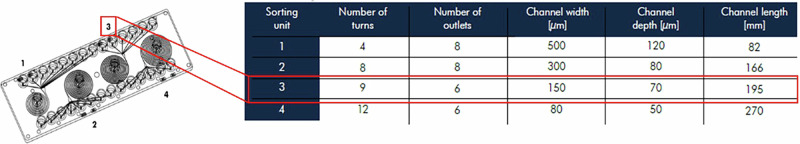
Schematic of the Spiral Sorter Fluidic 382 model, showing the four sorting units and an accompanying table with key details. Schematic drawing of spiral sorter Fluidic 382 model with four sorting unit^46^.

First, we simulated the steady-state flow of an aqueous fluid in the channel reported on the left in Fig. 4, which mimics the microfluidic chip identified by number 3 in the table in Fig. 6. The mathematical model of the system is composed of the steady-state mass and momentum balance equations for an incompressible Newtonian liquid with density of 1000 kg/m3 and viscosity of 0.001 Pa.s. Those are supplied with appropriate boundary conditions, i.e., a flow rate of 150 microliter/min at the inlet section of the channel (identified by the red arrow in Fig. 4), no-slip/no-penetration of the fluid at the lateral walls of the spiral channel, and an atmospheric pressure outflow condition at the outlet section of the channel. The 6 channels that branch off from the spiral channel to the collection wells of the microfluidic device were not considered in the simulation. In the right part of Fig. 4, we display the map of the velocity magnitude of the fluid in the cross section of the channel identified by the red dashed line in the left part of the figure, showing the well-known parabolic profile typical of the laminar flow of Newtonian liquids (the characteristic Reynolds number for this system is about 17). In addition, the vector field of the in-plane fluid velocity is also displayed, highlighting the presence of a cross-flow velocity directed towards the “inner” vertical wall of the channel, namely, the one closer to the centre of the spiral. After having simulated the fluid motion field in the channel, we exploited the Particle Tracing for Fluid Flow package of COMSOL Multiphysics to simulate the dynamics of point-like particles with properties mimicking those of the cells used in the experiments. It must be pointed out that the so called “one-way coupling” approach was adopted to simulate the motion of the particles. In other words, each particle moves throughout the channel under the action of the drag force exerted by the fluid, which depends on the size and density of the particle, on the density and viscosity of the fluid, and on the local value of the fluid velocity in the absence of particles. In addition, when a particle approaches a wall of the channel, it also feels the Saffman lift force^39^. On the other hand, there is no influence by the particles on the fluid flow field and there are no interactions among the particles. Three “families” of particles were included in the simulations, having diameter, density, and relative numerosity similar to those of lymphocytes, granulocites, and blasts considered in the experiments (the values are reported in the top-right part of Fig. 5). For each particle group, a uniform spatial distribution was imposed throughout the inlet section of the channel, together with a bounce-back condition on the lateral walls of the spiral channel. On the top-left of Fig. 5, the distribution of the particles in the outflow section of the channel is shown. As the channels that branch off from the spiral channel to the collection wells of the microfluidic device are not considered in the simulation, the section is divided into six portions by uniformly spaced vertical lines, ideally corresponding to wells 1 to 6 going from right (inner wall) to left (outer wall). It can be readily observed that, because of the secondary flows, the particles are overall pushed towards the inner outlets. In particular, the distributions of the different particles in outlets 1 and 2 arising from simulations are computed and compared with those obtained experimentally, yielding a fair quantitative agreement between experimental and numerical results (see histograms in the bottom part of Fig. 5), which is particularly notable if we consider that the simulations involve many simplifications.

## Discussion

Spiral microfluidic devices have advanced cell separation by providing rapid, efficient, and label-free solutions, especially for blood analysis and disease monitoring. Their portability and minimal sample requirements make them highly suitable for point-of-care and extreme environments, representing a significant improvement over traditional cell separation technique^40^. These findings validate the potential of this device for advanced biomedical applications, particularly where portability, precision, and efficiency are critical. In conclusion, the results of the procedure for sorting blood components are presented and discussed. The fast-processing time and the ability to collect and sort blood populations and to detect possible pathological cells from a small patient blood volume would open in future to a broad range of biomedical applications, for example in aerospace field. Indeed, there is a great demand of new technologies to monitor the human health during space missions avoiding complex sample preparations and suppling reliable results without highly skilled personnel. Space environment introduces several different hazards (distance, confinement, hostile/closed environments, radiation, and microgravity) that have health risks and consequences that span multiple organ systems. The astronauts are subjected to psycho-physical stress and possible inflammatory states. The knowledge gained in the human spaceflight field has highlighted the importance of frequent monitoring of astronaut’s health. Early diagnoses and in situ medical interventions could mitigate the risk of health consequences induced by space exposure^12^.

The successful adaptation of a commercial spiral microfluidic chip for the isolation of pathological blood cells underscores the strong translational potential of this platform. Its compatibility with unprocessed or minimally processed blood, minimal reagent use, and compact, self-contained operation aligns well with the operational constraints of point-of-care diagnostics—both in terrestrial and extraterrestrial settings. In particular, the ability to isolate leukemic blasts with high efficiency from peripheral blood without the need for antibody labeling or bulky instrumentation opens new possibilities for rapid, field-deployable diagnostics in resource-limited clinics, mobile health units, and remote environments^41^. In the context of human spaceflight, this approach offers a viable path toward in situ biomedical monitoring, enabling frequent immune surveillance and early detection of radiation-induced hematological abnormalities. The methodology presented here may thus serve as a foundation for the development of future integrated diagnostic systems for personalized health monitoring during long-duration missions and may inform broader applications in oncology, emergency medicine, and military healthcare systems. These results highlight that the microfluidic device is suitable for separating pathological cells from healthy ones in a sample with acute myeloid leukemia. Specifically, the sorting efficiency in well 2 represents a promising outcome for medical diagnostics, particularly for applications requiring the rapid separation of pathological cells in challenging environments, such as space settings. Although, the presented results highlight the strong potential of spiral microfluidic chips for point-of-care diagnostics, some limitations must be acknowledged. First, biological variability across donor samples may impact sorting performance and requires a broader validation. Second, although preliminary data show good agreement with simulations, reproducibility across different operators, devices, and environments—including microgravity—remains to be fully assessed.

In fact, our current work focused on demonstrating the feasibility and performance of the spiral microfluidic chip under terrestrial laboratory conditions. However, it is important to note that in our numerical simulations, gravitational effects were not included, as the governing hydrodynamic model does not include gravity forces in the momentum balance equation. The strong agreement observed between the simulated and experimental results (relative error < 1%) represents the proof of principle of the device performance and suggests its potential suitability for microgravity environments. Future work will involve experimental validation of chip performance in microgravity and parabolic flight environments to further assess its operational reliability in space-like conditions. Third aspect although the present setup employs a benchtop syringe pump (PHD Ultra) and manual collection, future developments will focus on miniaturizing and automating the system to make it fully compatible with space operation requirements. In particular, the integration of miniaturized capillary-driven or piezoelectric micro-pumping modules, combined with automated cell recovery through microvalve control or centrifugal microfluidic platforms, could significantly reduce the system’s size, weight, and power consumption. These modifications would enable the development of a compact, low-power, and autonomous bioanalytical platform suitable for continuous health monitoring in space and other resource-limited environments.

Finally, direct comparisons with conventional methods such as FACS are needed to benchmark the system’s sorting purity, diagnostic speed, and overall reliability. Addressing these aspects will be essential for future deployment in both space missions and terrestrial remote settings.

To further enhance diagnostic capabilities, future work may explore modifications to the chip design and its integration with optical, electrical or smart sensors^42^, enabling automated, real-time detection and quantification of sorted cell populations. Addressing these aspects will be essential for future deployment in both space missions and terrestrial remote settings^43–45^.

## Methods

### Spiral chip

Spiral sorter chip fluidic 382 (microfluidic Chip Shop – product code 10001825) was used for the experiments. Blood samples were tested with a specific chip structure (structure number 3) with 9 number of turns, 6 number of outlets, channel width of 150 μm and a channel depth of 70 μm. Each sorting unit possesses one inlet to introduce the fluid stream and six to eight outlets to collect sorted particle fractions. In- and outlets come in Mini Luer format and are interfaced with Mini Luer fluid connectors (Chip Shop product code 10000064). Silicon tubing (Chip Shop product code 10000033) with an inner diameter of 0.5 mm and outer diameter of 2.5 mm were using for the experiments. Spiral chips can be used to separate particles according to their size due to the so-called Dean forces. Channel dimension, number of spirals and diameter of the curvature influence the sorting effect. The sample is introduced with a syringe of 1 ml using a round-tip needle. through a central inlet and fractions with cells of different size can be received at the different outlet ports. The chip contains four sorting structures with the following parameters reported in Fig. 6.

### Biological sample

Whole blood was obtained from healthy volunteers at Azienda Ospedaliera Universitaria Federico II. Blood samples (3 mL) were collected in Vacutainer tubes (BD Biosciences, Japan) containing EDTA. After collection, the sample was used for experiments within one day. Consent for participation in the study was obtained in writing. All participants were enrolled using-approved protocols and provided informed consent. Experiments on healthy controls were performed using samples from five independent donors (*N* = 5), and for each experimental condition three technical replicates were carried out to ensure reproducibility, for a total of fifteen measurements. For the healthy sample, blood sample preparation consists of isolation of white blood cells (WBCs); dilution 5 ×10^5^ cell/ml^46^. Then, RBCs were diluted at final concentration of 5 × 10^5^ cell/ml. The inlet solution consisted of the two cells mixture (WBC, RBC) with equal cell concentrations (500.000 × 2 cells/mL). Infusion of sample in the sorting unit 3 with flow rate 150 μl/ml using PHD ultra syringe pump, Harvard apparatus, Volume loading: 1 ml. Cells recovery from the 6 outlets with a Gilson. After recovery, cell staining with APC/cyanine 7 anti-human CD 45 for WBCs and CD 235a - FITC (Glycoforin A) for RBCs was performed. In the end the cytofluorimetric cell analysis was performed using BD FACS Canto II. For the leukemia condition, analyses were conducted on three patient samples, with a comparable leukocyte count, each of which was analysed in triplicate to minimize experimental variability. For the pathological sample, that shows 8% lymphocytes, 83% blasts, and 5% granulocytes and 3% platelets with 100.000 WBC/μl, 50 μL of peripheral blood obtained from a patient with AML (LM5) was incubated with 4 ml of RBC lysis buffer [BD Pharm Lyse Lysing Buffer, Product no. 555899] according to the manufacturer’s procedure. After 2 wash steps with 1 mL of FACSFlow (BD FACSFlow Sheath Fluid Catalog No. 342003), WBCs pellet was resuspended in flow cytometry buffer and incubated for 15 min at 4 °C in the dark with fluorochrome-conjugated antibodies targeting specific cell surface markers: 5 μl of CD 56-PC 7 (BD, A21692) for blasts detection, and CD 45 – FITC (BD, Catalog No:345808) for all WBCs. The stained cells were resuspended in 10 mL of PBS and were sorted using spiral chip. Sorted cells were collected in suitable collection tubes and analysed with BD FACS Canto II flow cytometer.

### Syringe pump

PHD ULTRA Series MA1 70-3 syringe pump (Harvard apparatus) was used for the experiments. We select a flow rate of 150 μl/min related to the sorting unit 3, according to the spiral chip datasheet.

### Experimental separation efficiency

Separation efficiency refers to the recovery efficiency, defined as the percentage of target cells correctly collected in the expected outlet relative to the total number of target cells introduced into the device. Specifically, the separation efficiency (%) was calculated according to Eq. (1):

1 $${Separation\; efficiency}=\frac{{N}_{{target},{outlet}}}{{N}_{{target},{total}}}x\,100$$

### Calculation of percentage difference between experimental and numerical cell separation efficiencies

The percentage difference between experimental and numerical cell separation efficiencies was calculated to assess the agreement between the two approaches. The percentage difference was determined using Eq. (2):

2 $$\frac{\left({Experimental\; Value}-{Simulated\; Value}\right)}{{Experimental\; Value}\,}\,x\,100$$

Experimental tests yielded a separation efficiency of 83%, while numerical simulations resulted in an efficiency of 82.4%. Substituting these values in Eq. (2), it becomes Eq. (3):

3 $$\frac{\left(83-82.4\right)}{83\,}\,x\,100=0.72\, \%$$

This comparison allowed for quantitative evaluation of the simulation’s predictive accuracy relative to experimental outcomes, as recommended in recent studies validating microfluidic separation devices through both experimental and numerical methods.

### Statistical analysis

All results related to the cell sorting of WBCs and RBCs for the healthy sample, and blasts and leucocytes for the leukaemia sample, are presented as mean ± standard deviation (SD). Statistical significance was determined using one-way analysis of variance (ANOVA). When the ANOVA yielded a significant effect, pairwise comparisons were conducted using Welch’s *t*-test due to unequal variances. The statistical analysis conducted in this study was based on the approach described by D’Auria et al^47^. Groups that are not statistically different share the same letter, whereas distinct letters (a, b, c) indicate significant differences with a *p* < 0.05 (see Supplementary information).

## Supplementary information

Supplementary Information

## Data Availability

The datasets generated during the current study are available from the corresponding author on reasonable request.
