# Supplementary material for: Label-free blood cell separation for space health monitoring using a portable blast cell biochip
Source: NPJ Microgravity. 2026 Jan 27;12:17. doi: 10.1038/s41526-026-00561-9 (PMC12921280; doi:10.1038/s41526-026-00561-9)
Supplement: Supplementary file 1 — Supplementary Information [file 41526_2026_561_MOESM1_ESM.docx]

**Supplementary information**

**Statistical analysis** All results related to the cell sorting of WBCs and RBCs for the healthy samples, and blasts and leucocytes for the leukaemia samples, are presented as mean ±standard deviation (SD). Statistical significance was determined using one-way analysis of variance (ANOVA). When the ANOVA yielded a significant effect, pairwise comparisons were conducted using Welch’s t-test due to unequal variances. Groups that are not statistically different share the same letter, whereas distinct letters (a, b, c) indicate significant differences at p < 0.05.

| **ANOVA**  **& F-test** | **Groups** | **RBC** | | | **WBC** | | |
| --- | --- | --- | --- | --- | --- | --- | --- |
|  |  | **Outlet 1** | **Outlet 2** | **Outlet 3** | **Outlet 1** | **Outlet 2** | **Outlet 3** |
|  | **Average** | 13,24 | 63,40 | 84,60 | 91,15 | 34,13 | 13,32 |
|  | **S.D.** | 6,51 | 6,50 | 4,61 | 5,12 | 8,40 | 5,21 |
|  | **Count** | 15 | 15 | 15 | 15 | 15 | 15 |
|  | **Variance** | 42,36 | 42,28 | 21,25 | 26,21 | 70,53 | 27,11 |
|  | **Statistic** | **F** | **df (Between; Within)** | **p-value** | **F** | **df (Between; Within)** | **p-value** |
|  |  | 190.20 | 2; 42 | ≈ 0 | 196.67 | 2; 42 | ≈ 0 |

| **t-test** | **RBC** | | | **WBC** | | | **Statistic** | | |
| --- | --- | --- | --- | --- | --- | --- | --- | --- | --- |
|  | **OUT 1** | **OUT 2** | **OUT 3** | **OUT 1** | **OUT 2** | **OUT 3** | **t** | **df** | **p-value** |
|  | x | x |  |  |  |  | -12,19 | 7 | < 0.0001 |
|  | x |  | x |  |  |  | -20,01 | 7 | < 0.0001 |
|  |  | x | x |  |  |  | -5,95 | 7 | 0,0003 |
|  |  |  |  | x | x |  | 12,96 | 6 | < 0.0001 |
|  |  |  |  | x |  | x | 23,83 | 7 | < 0.0001 |
|  |  |  |  |  | x | x | 4,71 | 6 | 0,0016 |
|  | x |  |  | x |  |  | -21,04 | 7 | < 0.0001 |
|  |  | x |  |  | x |  | 6,16 | 7 | 0,0002 |
|  |  |  | x |  |  | x | 22,92 | 7 | < 0.0001 |

| **ANOVA**  **& F-test** | **Groups** | **BLASTS** | | | **LYMPHO** | | |
| --- | --- | --- | --- | --- | --- | --- | --- |
|  |  | **Outlet 1** | **Outlet 2** | **Outlet 3** | **Outlet 1** | **Outlet 2** | **Outlet 3** |
|  | **Average** | 72,83 | 83,23 | 57,58 | 9,77 | 8,18 | 4,96 |
|  | **S.D.** | 3,75 | 5,35 | 8,38 | 0,50 | 0,56 | 0,72 |
|  | **Count** | 9 | 9 | 9 | 9 | 9 | 9 |
|  | **Variance** | 14,08 | 28,66 | 70,27 | 0,25 | 0,31 | 0,52 |
|  | **Statistic** | **F** | **df (Between; Within)** | **p-value** | **F** | **df (Between; Within)** | **p-value** |
|  |  | 13.25 | 2; 24 | 0,0063 | 49.95 | 2; 24 | 0,0002 |

| **t-test** | **BLASTS** | | | **LYMPHO** | | | **Statistic** | | |
| --- | --- | --- | --- | --- | --- | --- | --- | --- | --- |
|  | **OUT 1** | **OUT 2** | **OUT 3** | **OUT 1** | **OUT 2** | **OUT 3** | **t** | **df** | **p-value** |
|  | x | x |  |  |  |  | -2,76 | 3 | 0,0352 |
|  | x |  | x |  |  |  | 2,88 | 2 | 0,0413 |
|  |  | x | x |  |  |  | 4,47 | 3 | 0,0104 |
|  |  |  |  | x | x |  | 3,68 | 3 | 0,0174 |
|  |  |  |  | x |  | x | 9,49 | 3 | 0,0012 |
|  |  |  |  |  | x | x | 6,11 | 3 | 0,0044 |
|  | x |  |  | x |  |  | 28,85 | 2 | 0,0006 |
|  |  | x |  |  | x |  | 24,15 | 2 | 0,0009 |

Cells flowing through the spiral microchannel exhibited the expected size-dependent lateral migration driven by Dean drag forces. As the suspension traveled along the curved geometry, larger cells (WBC and blasts) focused preferentially on the inner wall, whereas smaller cells (RBC) remained closer to the outer wall. This differential radial positioning resulted in a progressive stratification of the cell populations along the channel cross-section, ultimately producing distinct collection profiles at Outlets 1–3.

Consistent with this mechanism, a strong and statistically significant variation in cell concentration was observed across the three outlets for all four cell types (one-way ANOVA, p < 0.01 for every parameter; see Table 1). For RBC, the highest concentration was recovered in Outlet 3 (84.60 ± 4.61; group “a”), whereas Outlet 2 and Outlet 1 displayed progressively lower values (63.40 ± 6.50 and 13.24 ± 6.51; groups “b” and “c”). This distribution matches the expected behavior of smaller cells, which tend to migrate toward the outer streamline and are therefore directed to the distal outlet.

WBC displayed the opposite trend, with Outlet 1 showing the highest counts (91.15 ± 5.12; “a”), followed by Outlet 2 and Outlet 3 (34.13 ± 8.40 and 13.32 ± 5.21; “b” and “c”). This pattern reflects the preferential focusing of larger leukocytes near the inner wall and their subsequent extraction through the proximal outlet.

A similar size-dependent separation was observed for blasts and lymphocytes. Blasts, being larger, accumulated predominantly in Outlet 2 (83.23 ± 5.35; “a”) and Outlet 1 (72.83 ± 3.75; “b”), with significantly lower recovery in Outlet 3 (57.58 ± 8.38; “c”). Concerning Lymphocyte distribution: the uniform distribution across the wells reflects the low proportion of lymphocytes in the sample (~8% compared to 83% blasts). This pattern underscores the performance of the spiral microfluidic chip in isolating and concentrating larger pathological cells while maintaining the integrity of smaller, healthy cells. Overall, the outlet-specific profiles confirm that the spiral microchannel produces a robust, size-driven separation of hematological cell populations. The concordance between the known size hierarchy of the cells and their distribution across the outlets provides strong evidence of effective Dean-flow-mediated sorting.

**Cell viability**


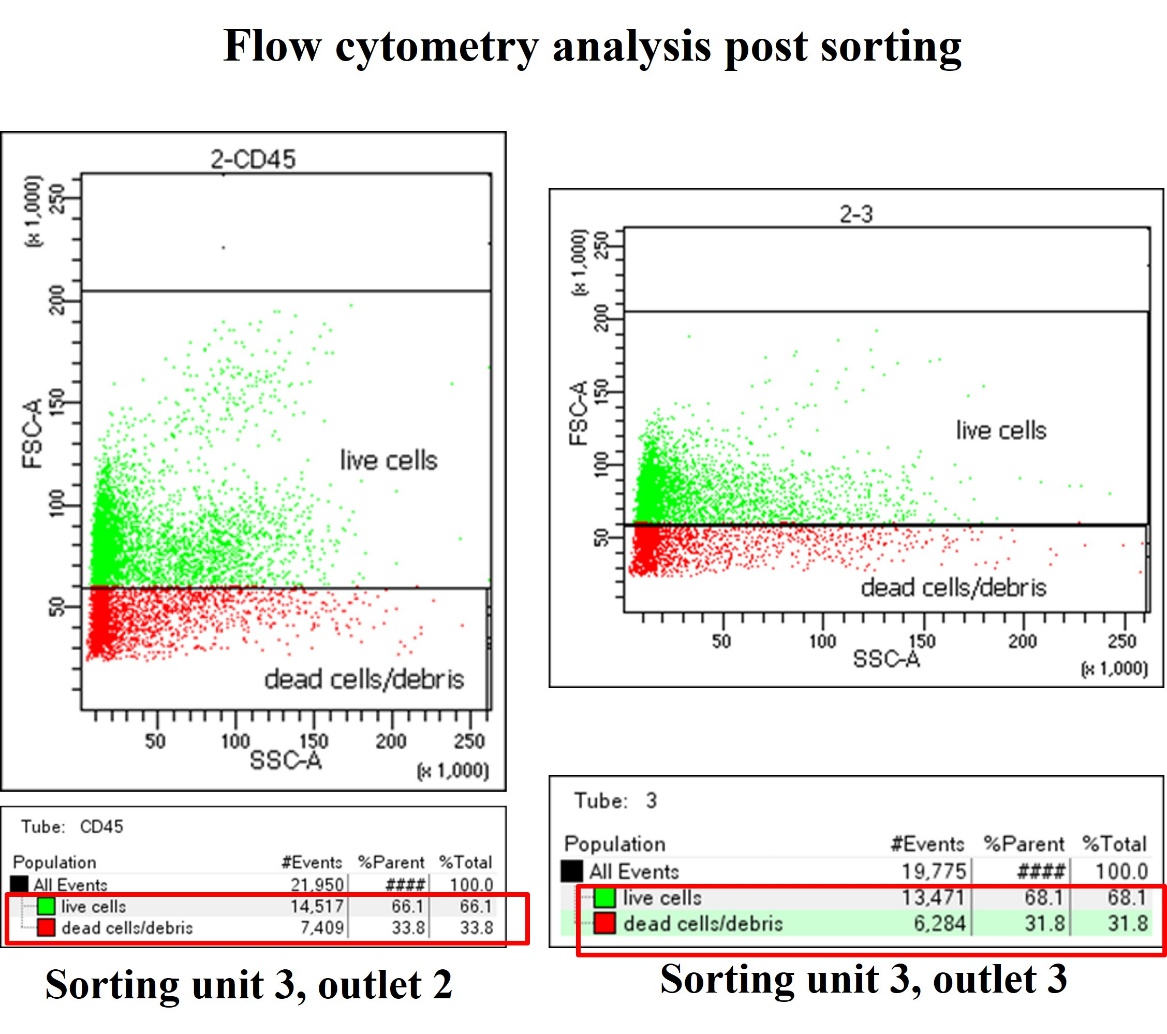


**Supplementary Figure S1**: Approximate evaluation of cell viability based on FSC/SSC parameters after microfluidic sorting.

In Figure S1, the representative post-sorting flow cytometry dot plots show the distribution of cells (healthy sample) based on forward scatter (FSC) and side scatter (SSC), reflecting cell size and internal complexity. The FSC/SSC profiles of the sorted samples closely match those of the expected populations, with no major shifts, indicating that the microfluidic separation process did not induce noticeable mechanical or structural damage. Although this morphological assessment provides only an indirect indication of viability, the quantitative analysis shows that approximately 60–68% of the cells fall within the live-cell gate, while about 30–34% are classified as dead cells or debris. These results support the mechanical gentleness and overall reliability of the sorting procedure.


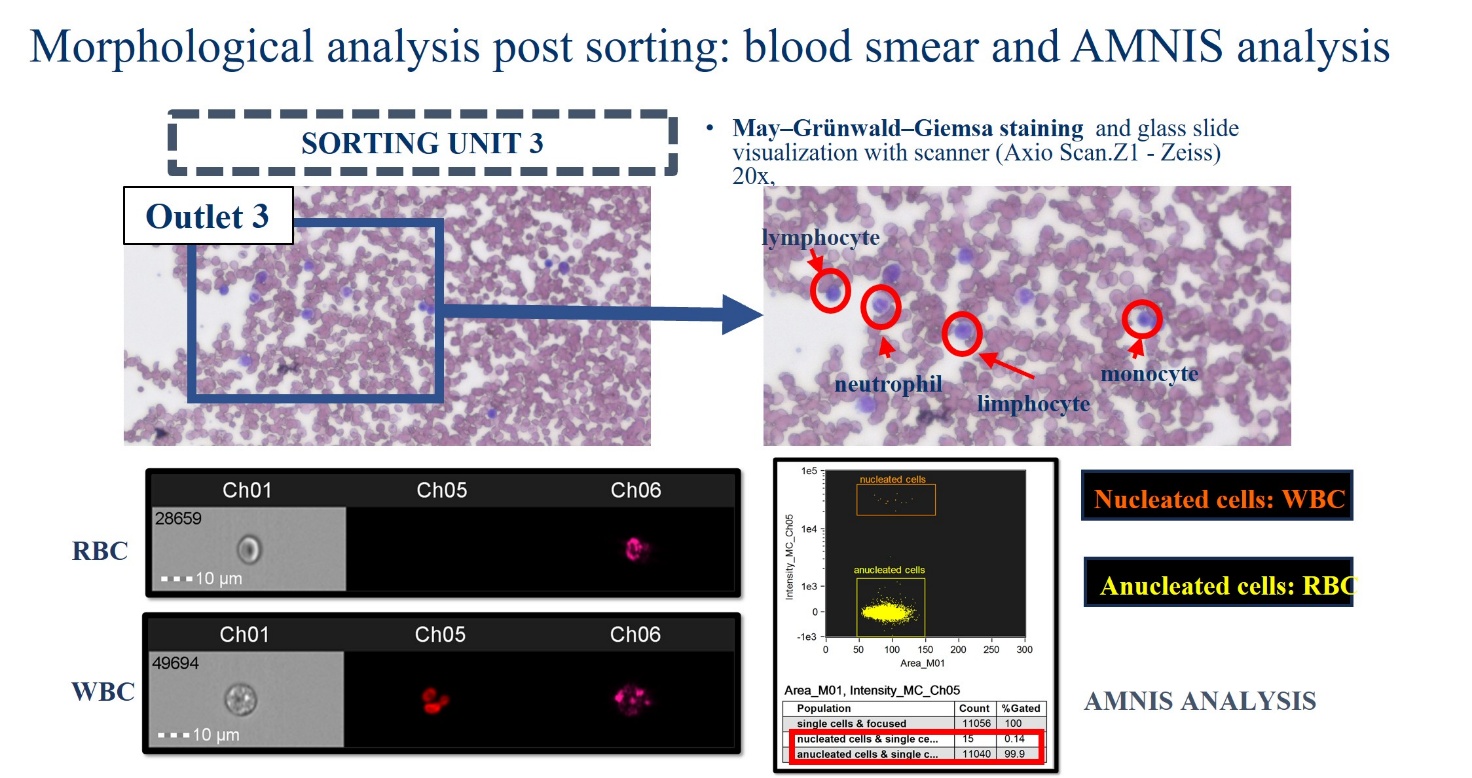


**Supplementary Figure S2**: Morphological assessment of post-sorting samples using blood smear staining and Amnis ImageStream analysis.

To obtain an approximate assessment of cell integrity and morphology after sorting, we performed a May–Grünwald–Giemsa staining on blood smears derived from Outlet 3. The stained slides allowed the visualization of the main leukocyte subpopulations, including lymphocytes, neutrophils, and monocytes, confirming that the sorted sample preserved recognizable morphological features.

In parallel, an Amnis ImageStream analysis was carried out to further evaluate cellular integrity by distinguishing nucleated from anucleated cells. According to the manufacturer’s instructions, the nuclear dye DRAQ5 was used to selectively stain the nuclei of white blood cells. The resulting images and scatter plots clearly separated nucleated cells (WBCs) from anucleated cells (RBCs), supporting the morphological observations obtained from the blood smear.

Together, the staining and ImageStream analyses provide an approximate yet consistent indication of preserved cell morphology and structural integrity in the post-sorting sample.
